# The utility of MRI in predicting radiographic erosions in the metatarsophalangeal joints of the rheumatoid foot: a prospective longitudinal cohort study

**DOI:** 10.1186/ar2737

**Published:** 2009-06-22

**Authors:** Matthew L Mundwiler, Paul Maranian, Douglas H Brown, Jeffrey M Silverman, Daniel Wallace, Dinesh Khanna, James Louie, Daniel E Furst, Michael H Weisman

**Affiliations:** 1 North Suburban Rheumatologists, 9301 W. Golf Road, #205, Des Plaines, IL 60016, USA; 2 David Geffen School of Medicine at the University of California in Los Angeles, 1000 Veteran Avenue, Los Angeles, CA 90095, USA; 3 Landmark Imaging Medical Group, Inc., 11620 Wilshire Boulevard, #100, Los Angeles, CA 90025, USA; 4 Cedars-Sinai Medical Center, 8700 Beverly Boulevard, Los Angeles, CA 90048, USA

## Abstract

**Introduction:**

Magnetic resonance imaging (MRI) may reveal rheumatoid arthritis (RA) changes in the feet when hands are normal. The purpose of this study was to determine the sensitivity, specificity, positive predictive value (PPV), and negative predictive value (NPV) of a metatarsophalangeal (MTP) erosion on MRI to predict a subsequent radiographic erosion in the same joint. Similar analyses were performed for bone marrow edema, predicting a subsequent MRI erosion. Descriptive results of other lesions are reported.

**Methods:**

Fifty patients with RA of less than 5 years' duration who were rheumatoid factor-positive and/or anti-cyclic citrullinated peptide-positive were recruited. Patients on anti-tumor necrosis factor (TNF) therapy were excluded. Anti-TNF therapy could begin after enrollment. MRI and radiographs of the 3rd, 4th, and 5th MTP joints bilaterally were taken at baseline and at 6, 12, and 24 months. Clinical data were collected.

**Results:**

Fifty patients were recruited; 46 patients had suitable data. Results for MRI erosions predicting subsequent radiographic erosions for 6, 12, and 24 months, respectively, were as follows: sensitivity 0.75, 0.60, 0.75; specificity 0.93, 0.94, 0.94; PPV 0.086, 0.10, 0.17; NPV 0.998, 0.995, 0.995. Results for MRI bone marrow edema predicting MRI erosions at 6 and 12 months, respectively, revealed sensitivity 0.50, 0.67; specificity 0.97, 0.97; PPV 0.25, 0.50; NPV 0.99, 0.99. Synovitis was the most common finding and, when present in isolation, resolved on 67.3% of subsequent studies. MRI erosions persisted on subsequent studies with one exception. Forty-six percent of the cohort was on anti-TNF therapy after study inception.

**Conclusions:**

The PPV of MRI erosions to predict subsequent radiographic erosions was low. Similarly, the PPV of bone marrow edema to predict a later MRI erosion was low. Alternatively, the NPV of the absence of an MRI erosion or bone marrow edema predicts that a later radiographic erosion or MRI erosion will likely not develop. Anti-TNF therapies may have resulted in the lower-than-anticipated PPVs. MRI descriptions of bone edema may represent a more critical time to treat in order to avoid damage, whereas an MRI erosion represents more permanent damage. This study suggests that imaging modalities more sensitive than radiographs are necessary to monitor disease in the biologic era.

## Introduction

It is becoming axiomatic that diagnosing and treating rheumatoid arthritis (RA) before radiographic damage and functional disability occur are desirable [1]. Therefore, it would be beneficial to employ non-invasive tests to reliably predict if and when this radiographic damage might take place. Magnetic resonance imaging (MRI) may fulfill this need. An earlier study by Forslind and colleagues [2] revealed that MRI images from RA patients demonstrate several positive findings even when conventional radiographs are normal. Other investigations demonstrated that erosions on radiographs were evident on MRI a median of 2 years earlier [3] and that MRI could be a superior tool for tracking progression of RA in patients [4]. Brown and colleagues [5] recently showed that evidence of inflammatory activity on MRI persists despite the appearance of clinical remission. To date, most of the MRI-related research has focused on the hand and wrist; however, Boutry and colleagues [6] demonstrated that MRI findings in the feet were as prevalent as hand findings in early-RA patients. Another study showed that early-RA patients may have MRI findings in the feet when the hand studies are normal [7].

As a result of the above considerations, we chose to concentrate on the feet of RA patients for our observations. The primary objective of this research was to determine the sensitivity, specificity, and predictive values of MRI erosions in metatarsophalangeal (MTP) joints to predict new radiographic erosions in the same joint after 6, 12, and 24 months.

## Materials and methods

### Patient selection and recruitment

This study has a prospective longitudinal cohort design. Fifty RA patients with less than 5 years of disease duration were recruited from three centers in the Los Angeles area to undergo repeat imaging studies. The study protocol was approved by the institutional review boards of all study centers (Cedars-Sinai Medical Center, University of California at Los Angeles, and Harbor-University of California at Los Angeles). Written informed consent was obtained from all patients prior to their entrance into the study. The patients met the 1987 American College of Rheumatology classification criteria for RA, were either rheumatoid factor (RF)-positive or anti-cyclic citrullinated peptide (anti-CCP)-positive, and were at least 18 years old. Patients were excluded if they had been treated with an anti-tumor necrosis factor (anti-TNF) biologic prior to the start of the study, had a positive pregnancy test, or had any contraindications to MRI. Patients who met study criteria were followed for 2 years. To maximize the probability to detect new radiographic erosions attributable to RA and their preceding MRI findings, we chose to examine the 3rd, 4th, and 5th MTP joints. This decision was based upon previous work that indicated that the earliest radiographic erosions in RA would occur in these areas [8,9].

At the initial visit, bilateral foot radiographs and MRI of the 3rd, 4th, and 5th bilateral MTP joints were obtained. In addition, subject pain assessment, health assessment questionnaire disability index (HAQ-DI), RA severity scale (RASS), swollen and tender joint count, C-reactive protein (CRP), erythrocyte sedimentation rate (ESR), RF, anti-CCP, complete blood cell count, aspartate aminotransferase, alanine transaminase, alkaline phosphatase, and a urine pregnancy test were recorded. At the 6-month visit, MRI and radiographs of the forefeet were again taken as well as a pain assessment, RASS, swollen and tender joint count, and a urine pregnancy test. At the 1-year visit, MRI and radiographs of the bilateral forefeet were taken. At the 24-month visit, bilateral radiographs of the forefeet, subject pain assessment, HAQ-DI, RASS, and swollen and tender joint count were obtained. At all visits, a list of patient medications was recorded. Therapy for the patient's RA was at the discretion of the patient's physician. MRI results were not provided to the caregiver and did not influence management decisions.

### Protocol for magnetic resonance imaging of the bilateral forefeet

Images were obtained with a 1.5-Tesla whole-body MRI scanner (Signa Horizon, LX; General Electric Medical Systems, Milwaukee, WI, USA) with and without gadolinium contrast enhancement (0.1 mmol/kg of gadopentetate dimeglumine; Magnevist; Berlex, now part of Bayer HealthCare Pharmaceuticals, Montville, NJ, USA) and an optimal field of view (FOV) for the 3rd, 4th, and 5th MTP joints to be taken together. Each MRI involved three sequences using a dual-TMJ coil (General Electric Medical Systems) with an internal diameter of 3 inches. First, a multi-slice, coronal T1-weighted spin-echo sequence (300/14 repetition time/echo time [TR/TE], slice thickness of 4 mm) was completed. Next, a multi-slice, axial fast inversion sequence (3,000/34 TR/TE, 130 inversion time, slice thickness of 3 mm) was obtained. Finally, pre- and post-contrast (Magnevist) multi-slice, axial fat-suppressed spoiled gradient echo-recalled sequences (150/2.8 TR/TE, flip angle 70 degrees) were completed. Additional imaging parameters were an FOV of 10 × 10 cm, matrix of 256 × 192, and two acquisitions.

### Protocol for radiographs of the bilateral forefeet

Radiographs (anterior-posterior, internal-oblique, and lateral films of the feet) were performed with a computed radiography system (Fuji System; Fujifilm Corporation, Tokyo, Japan). Average settings were 55 to 60 kV, 200 to 250 mA, and 2.5 mA. Adjustments were made depending on body habitus.

### Protocol for scoring of the forefeet magnetic resonance imaging

Joints scored were the bilateral 3rd, 4th, and 5th MTP joints of the feet. Findings in other structures were not documented. Scoring was based on version 3 of the fully validated RA MRI score (RAMRIS) reviewed at the OMERACT (outcome measures in RA clinical trials) 6 proceedings [10]. The same principles applied to scoring the hand films in those proceedings were applied to the readings of the MTPs for this study. Erosions were defined as bony defects with sharp margins, visible in axial and coronal views with at least one view showing a cortical break. The area of interest was the first 1 cm of subarticular bone at both the metatarsal and phalangeal bases of the individual MTP being scored. The actual score was based on the percentage of this area being eroded. If there was no erosion, the score was 0. If 1% to 10% of the area was eroded, the score was 1; 11% to 20% resulted in a score of 2; and 21% to 30% resulted in a score of 3; and so on up to a maximum score of 10. Each joint had a possible score of 20 since the maximum score of each base was 10.

A defect was considered the loss of trabecular bone without a visible cortical break; the same scoring principles used for erosions applied to defects. Bone marrow edema (BME) was defined as a high-intensity focus with ill-defined margins seen on T2-weighted sequences. Again, the area of interest was the first 1 cm of subarticular bone of the metatarsal and phalangeal bases. BME was also scored based on the percentage of involvement of this area in the same manner as erosions. If there was an erosion or defect present, BME scoring was based on the percentage of involvement of the remaining bone.

Synovitis was defined as synovial enhancement that appeared thicker than the width of the joint capsule after the administration of gadolinium. Possible scores were 0 to 3 for the joint, with 0 being no synovitis, 1 being mild synovitis, 2 being moderate synovitis, and 3 being severe synovitis. Because the new appearance of an erosion was the primary outcome of interest, only the scores were recorded. The dimensions of the lesions were not considered during this analysis.

### Protocol for scoring of the forefeet radiographs

Scoring was based on the Sharp/van der Heijde method [11], with the 3rd, 4th, and 5th bilateral MTPs being the joints of interest. Erosions were defined as a discrete area of bone loss. A score of 0 signified no erosion, 1 signified discrete erosion, 2 signified a larger erosion, and 3 signified an erosion covering more than 50% of the joint surface. The maximum score for each bone surface was 5, and the maximum score for one joint was 10. Joint space narrowing was scored as follows: 0 was no narrowing, 1 was focal, 2 was generalized with at least 50% of the joint space left, 3 was generalized with less than 50% of the joint space left, and 4 was ankylosis or complete subluxation. The maximum score for one joint was 4. Because the new appearance of an erosion was considered the primary outcome of interest, only the score was recorded. The dimensions of the lesions were not considered during analysis.

### Protocol for reading of all images

Each study was read by two musculoskeletal radiologists without knowledge of any clinical data; the studies were read in unison, and consensus was required. MRI images were read independently and without knowledge of radiographs, and each limb was read independently. Readers were blinded to the identity of the patient. MRI images and radiographs were read after the imaging studies were completed for each time period. Readings were performed at a dedicated reading workstation (General Electric Medical Systems).

### Magnetic resonance imaging erosions predicting subsequent radiographic erosions

The primary objective of this study was to determine the sensitivity, specificity, positive predictive value (PPV), and negative predictive value (NPV) of an MRI erosion to predict a new radiographic erosion in the same joint at a subsequent time point. The design of this study permitted the calculation of these parameters at 6, 12, and 24 months between the detection of the MRI erosion and the subsequent appearance of a new radiographic erosion.

It should be noted when determining the sensitivity, specificity, PPV, and NPV at 6 and 12 months that, if patients completed all studies, one joint could contribute two data points to each individual calculation. For example, the progression of a single MTP could be followed from the baseline MRI to the 6-month radiograph and from the 6-month MRI to the 12-month radiograph. This strategy was considered appropriate for two reasons. First, the age of any MRI erosion observed at baseline was unknown. MRI erosions at baseline were considered equivalent whether or not they were new or had been present for some time. Second, this strategy enabled us to include new MRI erosions occurring during the study after baseline.

### Bone marrow edema predicting magnetic resonance erosions

The sensitivity, specificity, PPV, and NPV of bone edema predicting a new MRI erosion in the same joint 6 and 12 months later were calculated in the same manner as outlined above. If an MRI erosion was already present when the BME was first noted, the joint was not included in the analysis.

### Descriptive data

The following calculations were made for each MRI finding (synovitis, BME, bone defects, and bone erosions): (a) The percentage of the patients completing more than one study (n = 46) having each MRI finding at least once. (b) The percentage of the 276 joints studied (46 patients × 6 joints) having each finding at least once. (c) The number of times a finding was observed if each joint on each MRI is considered separately. For example, consider one patient having three joints imaged twice. If two joints had BME on the first image and one joint had BME on the second image, then BME would have been observed three times. (d) The frequency of other findings when the finding of interest is present. Again, consider one patient having three joints being imaged twice. If one joint had an erosion on the first study and two joints had an erosion on the second, then erosions would have been observed three times. If synovitis was seen with the first joint on the first study but was not seen with the two erosions on the second study, synovitis would have been observed with erosions 33% of the time. (e) The fate of each finding on subsequent MRIs. Because each patient completed different studies at different time intervals and the initial appearance of each finding varied widely, time intervals were not considered as part of the analysis. A persistent finding, however, had to be present for at least 6 months as that was the minimal interval between MRI studies.

### Statistical methods

#### Comparison of baseline differences between subjects

Baseline differences between the five groups are summarized in Table 1 with respect to clinical measures such as ESR, CRP, disease activity score using 28 joint counts (DAS28), HAQ, swollen and tender joint counts, physician global assessment, and disease duration. Data analyses were explored using a combination of global tests (analysis of variance and Kruskal-Wallis, as appropriate) and pairwise comparisons controlling the experiment-wise error rate (Tukey's honestly significant differences test).

**Table 1:** Magnetic resonance imaging (MRI) erosions predicting subsequent radiographic erosions and MRI bone marrow edema predicting subsequent MRI erosions

MRI erosions predicting subsequent radiographic erosions										
Time interval between studies	Patients	New x-ray erosions preceded by MRI erosion	MRI erosions NOT leading to x-ray erosion	New x-ray erosion NOT preceded by MRI erosion	Studies not having an x-ray or MRI erosion	Sensitivity	Specificity	PPV	NPV	Odds ratio (CI)

6 months	43	3	32	1	430	0.75	0.93	0.086	0.998	^a^40.3 (4.08–398.7)
12 months	39	3	27	2	417	0.6	0.94	0.10	0.995	^a^32.2 (3.7–144.6)
24 months	41	3	15	1	218	0.75	0.94	0.17	0.995	^a^43.6 (4.27–445)

MRI bone marrow edema predicting subsequent MRI erosions										

Time interval between studies	Patients	New MRI erosions preceded by BME	BME NOT leading to MRI erosion	New MRI erosion NOT preceded by BME	Studies not having an MRI erosion or BME	Sensitivity	Specificity	PPV	NPV	Odds ratio (CI)

6 months	43	4	12	4	410	0.50	0.97	0.25	0.99	^a^34.17 (7.62–153.1)
12 months	39	6	6	3	204	0.67	0.97	0.50	0.99	^a^68.0 (13.6–338.9)

The data for the major endpoints of the study are summarized here. The number of patients at each time interval varies because not all patients completed all studies. All radiographic erosions not preceded by an MRI erosion were preceded by MRI defects. There were only five new radiographic erosions for the entire cohort. ^a^Statistically significant. BME, bone marrow edema; CI, confidence interval; NPV, negative predictive value; PPV, positive predictive value.

#### Calculation of sensitivity, specificity, positive predictive value, and negative predictive value

For the purposes of the present study, a study can be represented as an ordered pair ('a, b'), where 'a' takes on the value 1 or 0 based on whether or not for a given joint an erosion appears on MRI at time 1, and 'b' takes on the value 1 or 0 based on whether or not an erosion appears on radiograph at time 2. When the time interval of interest is 6 months, this gives rise to a possible total of 12 studies per subject, there being 6 joints per subject and two 6-month intervals represented in the data: baseline (time 1) to 6 months (time 2), and 6 months (time 1) to 12 months (time 2). When the time interval of interest is 12 months, the number of studies per subject is also 12 (indicating two 12-month intervals), while for the 24-month interval, the number of studies per subject is 6 (only one 24-month interval is possible). Based on the total number of studies, 2 × 2 classification tables were generated for each time interval of interest (6 months, 12 months, and 24 months) and used to determine the sensitivity, specificity, PPV, NPV, and odds ratio (OR) for the presence of radiographic erosions relative to the prior appearance of erosions on MRI. The same method was used to generate tables for examining the relationship between BME and their predictive value for MRI erosions. Computations were achieved by using the statistical software packages SAS System Release 9.1.3 (SAS Institute Inc., Cary, NC, USA) and Stata/SE 9.2 (StataCorp LP, College Station, TX, USA).

## Results

### Patients

**Figure 1:**
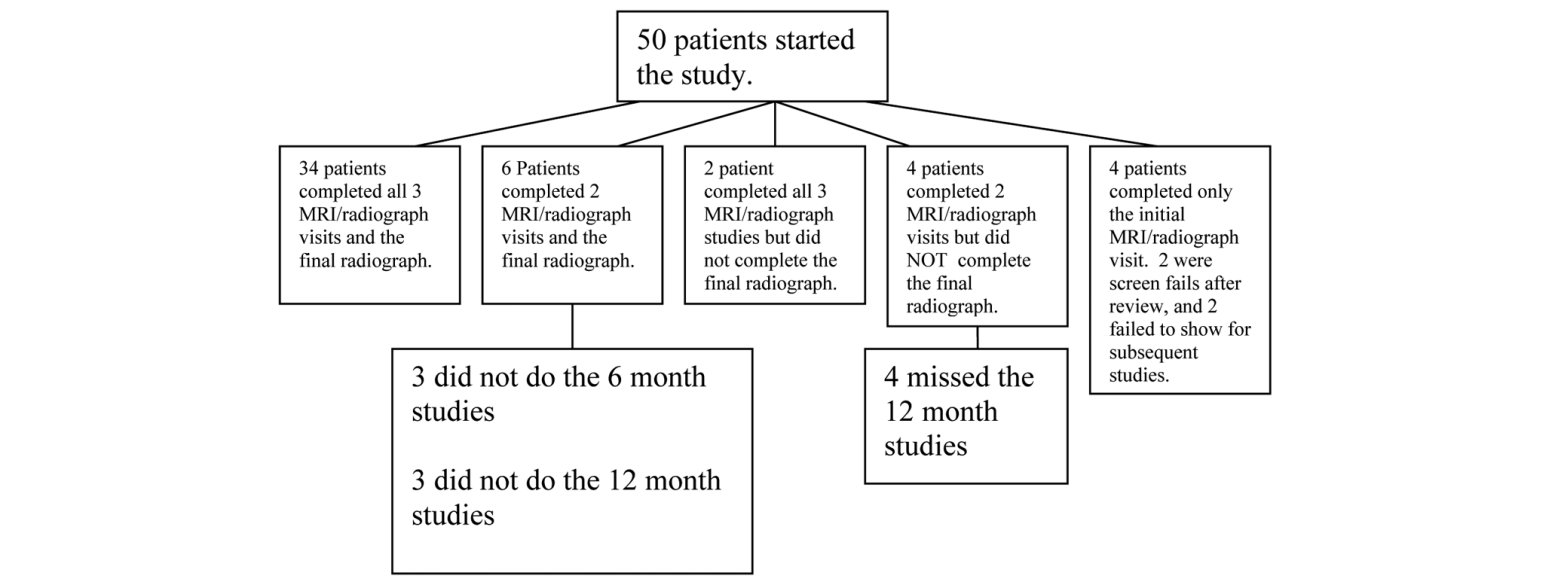
Studies completed by cohort. All 50 patients underwent the initial magnetic resonance imaging (MRI) and radiograph. Patients who completed only two MRI/radiograph studies underwent the initial studies and either the 6-month or 12-month study.

Fifty patients were recruited; 46 patients completed at least one study beyond baseline and 34 patients completed all the protocol-scheduled studies. Figure 1 indicates which studies were or were not completed by the cohort. Of the 46 patients who completed at least one study, 36 were female, 21 were placed on an anti-TNF agent during the study, 17 were on methotrexate or leflunomide without a biologic, 37 were RF-positive, and 31 were anti-CCP-positive; the average disease duration was 1.34 years. The following subgroups were compared within the cohort: patients with no MRI or radiographic findings, patients with synovitis on MRI only, patients with edema or defect or erosion on MRI, patients with erosions on MRI, and patients with radiographic findings. Although there was no pre-specified stratification, there were no differences between groups in regard to disease duration, biologic use, methotrexate use, or the following parameters at baseline: ESR, CRP, DAS28, HAQ, swollen joint count, tender joint count, and physician global assessment.

### Magnetic resonance imaging erosions predicting radiographic erosions

The data for MRI erosions predicting subsequent radiographic erosions are summarized in Table 1. For all time intervals studied, the PPV of an MRI erosion for a later radiographic erosion was low (6-month = 0.086, 12-month = 0.10, 24-month = 0.17). On the other hand, the high NPV indicates that the absence of an MRI erosion almost guarantees that a radiographic erosion will not form over the time intervals studied (6-month = 0.93, 12-month = 0.94, 24-month = 0.94). Having an MRI erosion, however, dramatically increases the chance of the formation of a radiographic erosion as indicated by the ORs (6-month = 40.3, 12-month = 32.2, 24-month = 43.6). We caution, however, that confidence intervals (CIs) were constructed without taking into account the hierarchical nature of the data and therefore are likely to underestimate the standard error of the OR estimates. The number of new radiographic erosions was much lower than we anticipated, perhaps because many patients used anti-TNF agents.

Despite the increased sensitivity of MRI to pick up bone lesions, we were surprised to find that the sensitivity of MRI for detecting radiographic erosions at a later time was 0.60 to 0.75 in our study. When this issue was explored, the radiographic lesions not preceded by an erosion were preceded by a bone defect, a bony lesion without a cortical break. If defects were taken into account, the sensitivity of this combined finding on MRI would be 1.00.

### Bone marrow edema predicting magnetic resonance erosion formation

Table 1 also summarizes the results for BME predicting MRI erosions. BME has a low PPV for predicting an MRI erosion (6-month = 0.25, 12-month = 0.50), but its absence makes the formation of an MRI erosion over the course of 1 year highly unlikely as indicated by the high NPV (6- and 12-month = 0.99). Having BME, however, dramatically increases the chance of an MRI erosion to form as indicated by the ORs (6-month = 34.17, 12-month = 68.0). As is the case for MRI erosions, the large CIs indicate that caution is required when using BME as a predictor in an individual patient.

### Descriptive data

Table 2 summarizes the frequency of BME, bone defect, bone erosion, and synovitis on MRI by the absolute number of times each was observed, by patient, and by joint. Also reported is the percentage of time the other findings are present when the finding of interest is taken into account. Synovitis was the most common finding in the study and was often accompanied by the other findings. BME was accompanied by synovitis in all but one instance. Next, we analyzed the resolution and persistence of each lesion. The lesion of interest had to be present and a subsequent MRI of that joint had to be completed for this analysis. Among 24 MTPs with appropriate data, 70.8% of BME resolved and 28.2% persisted on the subsequent studies. We then assessed bone defects and bone erosions on MRI. Because the actual dimensions of each lesion were unknown, we could analyze only those lesions that occurred without the other being present. For example, we could analyze an erosion only if there was no defect. Bone defects were an isolated finding 28.8% of the time. Among 16 MTPs with appropriate data, 31.1% of these defects persisted without change on subsequent studies, 37.5% completely resolved on the subsequent studies, and 31.1% were followed by the development of bone erosions on subsequent studies. Bone erosions were an isolated finding 17.1% of the time. Twenty-eight MTPs had a bone erosion and a subsequent MRI; 96% of these erosions persisted on subsequent studies, and only one small erosion resolved on a subsequent MRI.

**Table 2:** Summary of secondary endpoints: descriptive statistics

	Bone marrow edema	Defects	Erosions	Synovitis
Patients with this finding at least once during the study, number (percentage)	16 (34.8%)	13 (28.3%)	16 (34.8%)	44 (73.9%)
Joints with this finding at least once during the study, number (percentage)	28 (10.1%)	24 (8.7%)	30 (10.9%)	102 (37%)
Number of times the finding was observed	45	45	70	174
Percentage of time the finding was observed with bone marrow edema	-	26.7%	35.7%	21.8%
Percentage of time the finding was observed with defects	26.7%	-	20%	14.4%
Percentage of time the finding was observed with erosions	60%	31.1%	-	33.9%
Percentage of time the finding was observed with synovitis	91.1%	64.4%	74.1%	-

The 46 patients who completed more than one study are considered here. Two hundred seventy-six joints were imaged by magnetic resonance imaging (MRI) at least twice. A single joint was considered to have the finding if it appeared once in that joint during any of the MRI studies. The total number of times each characteristic was found was also counted, as was the percentage of the time each finding occurred with another. For examples of how these data were calculated, please refer to items (c) and (d) of the Descriptive data section of Materials and methods.

Synovitis was usually present with other lesions but was an isolated finding 29.9% of the time. Synovitis was detected as an isolated finding in 52 MTP joints that had subsequent studies. On subsequent studies, 67.3% of the isolated synovitis resolved without the appearance of other lesions and 26.9% persisted without other lesions appearing. Five point eight percent of MTPs with synovitis as an initial isolated finding developed bony (defect or erosion) lesions. The synovitis persisted in two instances and resolved in one instance when these bony lesions appeared.

### Therapeutic effects

Because there were few new radiographic types of erosion, we proceeded to determine whether therapies had an effect. We could not accurately relate the timing of therapies (started in an uncontrolled or unrecorded fashion) to the occurrence of radiographic erosions. No distinct pattern relating new radiographic erosions to therapy received during the study emerged (data not shown). We also attempted to determine whether therapies had an impact on MRI findings and chose to base this analysis on bone erosions because these lesions are persistent on subsequent studies. The reversibility of the other lesions did not allow meaningful analysis since we could not relate the timing of therapies to the timing of the MRI studies. When we compared the initial and final RAMRIS erosion scores of patients on different therapies, we found that the majority of patients with worsening erosion scores were on anti-TNF therapy, whereas the patients on methotrexate or leflunomide usually had stable or improving scores. This numerical difference may reflect selection bias, as patients with more active disease (and more likely to show active disease on MRI) were probably placed on anti-TNF agents (data not shown).

## Discussion

The major finding in this study was that detection of an erosion in the MTP joints of an RA patient by MRI does not have a high predictive value for subsequent formation of a radiographic erosion in the same joint over a 2-year time period. Despite our focus on the joints that are believed to be most likely to erode in an RA patient [12], the number of new radiographic erosions was low in the face of numerous MRI erosions in our study (87 total MRI erosions as compared with 5 new radiographic erosions). The low number of radiographic findings was surprising given the risk of erosive disease expected in a population selected for RF and/or anti-CCP positivity.

Three well-done similar studies of the rheumatoid wrist (summarized in Table 3) reported more rapidly developing erosive lesions. Comparing these studies to ours might provide insight into these differences. Wrist images include approximately 18 potential articulations per patient and a larger potential surface area for erosion when compared with 6 MTP joints per patient. Also, two of these studies followed patients for longer periods of time, increasing the chances of seeing a subsequent radiographic erosion. Furthermore, there may be pathologic differences between the wrist joints and MTPs. Despite radiographic studies indicating that MTP erosions occur earlier in the disease course [8,9] and MRI studies indicating that MTP erosions are present on MRI when not present in the hand [7], few studies examine how fast MRI erosions progress to radiographic erosions in the MTPs. More evaluations that have a design similar to ours would be necessary to draw accurate conclusions. Histologic studies that include comparisons with wrist studies would be necessary to determine pathologic reasons.

**Table 3:** Summary of similar studies

Study	Results summary
Østergaard *et al*. [3] (wrist)	Most radiographic erosions were evident 2 years prior on MRI. ^a^The PPV of MRI erosion predicting radiograpic erosion over the course of 5 years was estimated to be 52%. The relative risk of developing radiographic erosion when MRI erosion is present is 4.1 after 5 years.
Scheel *et al*. [23] (wrist)	Forty-one percent of MRI erosions were seen on radiographs 7 years later. ^a^The estimated PPV of MRI erosion predicting radiographic erosion at 7 years is 50%. ^a^The estimated relative risk of MRI erosion for future radiographic erosion at 7 years is 43.
McQueen *et al*. [24] (wrist)	This is a study of early rheumatoid arthritis with symptoms of 6 months or less. Statistical results of an MRI erosion in the wrist predicting future radiographic erosion in the wrist at 1 year were sensitivity of 83%, specificity of 70%, PPV of 58%, NPV of 91%, and odds ratio of 11.6. Twelve new erosions over a 1-year period were seen in the dominant wrist of 42 patients.

All three of these studies featured the wrist. ^a^Estimated from data reported in the article. MRI, magnetic resonance imaging; NPV, negative predictive value; PPV, positive predictive value.

The radiologists' knowledge of the timing of the imaging studies being read could account for the large number or MRI erosions read in comparison with the number of radiographic erosions. Although our radiologists were blinded to the identity of the subjects and did not compare the image being read with other images from the same subject, they were aware at what time point in the study the image was taken. It is usually expected for patients with a longer disease duration to accumulate more damage. It is possible that the radiologists were more likely to be sensitive to small lesions, especially when reading images taken later in the study. Future investigations should be designed to specifically address this issue.

The disease durations of our subjects may also have contributed to the low number of new erosions seen. Approximately half of our cohort had a disease duration of less than 1 year at the start of the study, and a small percentage had a disease duration of up to 5 years. A study by McQueen and colleagues [13] demonstrated that the highest rates of erosive activity may occur within the first year of disease. Although there were only five new radiographic erosions in our study, six radiographic erosions were already present in our cohort at baseline. Limiting the cohort to patients with less than 1 year of disease may have increased our PPV of an MRI erosion predicting a subsequent radiographic erosion.

The use of anti-TNF agents also likely contributed to the low number of new radiographic erosions. Forty-six percent of our patients were on anti-TNF agents at some time during the study. It is now accepted that the anti-TNF agents are highly effective in suppressing radiographic damage even in the presence of some continued disease activity [14,15]. Furthermore, the use of anti-TNF inhibitors in the first 2 years of disease halts radiographic progression even more effectively [15]. When we evaluated the MRI findings in our study, we found that the patients with more severe erosive disease on MRI were being treated with anti-TNF therapies. Our study was not designed to directly assess the impact of treatments on the imaging outcomes, and the treatment data may reflect the selection bias that the most severe patients were placed on anti-TNF therapy during the study. To accurately assess the effects of anti-TNF agents on MRI outcomes, a study controlling for such therapy would need to be performed.

Despite the low number of new radiographic erosions, 10% of the joints imaged had MRI erosions. Furthermore, these erosions persisted on the subsequent MRI 96% of the time. Thus, the presence of an MRI erosion likely indicates more permanent damage and more severe disease. Ultimately, our data may indicate that imaging modalities more sensitive than radiographs are needed since regular use of biologics will suppress radiographic changes. MRI erosions, however, are readily visible even when a large percentage of the cohort are exposed to TNF inhibition.

BME is being accepted as an important lesion on MRI in RA because studies have indicated that it may predict future joint damage. McQueen and colleagues [16] elegantly showed that total BME score of the wrist predicted erosive progression on radiographs 6 years later. Haavardsholm and colleagues [17] and Hetland and colleagues [18] reproduced these findings at 1 and 2 years, respectively.

As opposed to analyzing total BME score, we wanted to determine whether BME predicts erosive changes in the same joint on MRI. BME predicted a 50% likelihood of a new bone erosion on MRI in the same joint over a 1-year period (PPV = 0.50, OR = 68.0 at 12 months, CI = 13.6 to 338.9); the absence of BME almost guarantees that a bone lesion would not develop on subsequent MRI over a 1-year period (NPV = 0.99). BME, however, seems to be a readily reversible lesion, resolving on 70.8% of subsequent studies. Recent histologic studies have touched on why this association between BME and joint destruction exists. Jimenez-Boj and colleagues [19] concluded that bone marrow infiltration by inflammatory cells may be secondary to small breaks in cortical bone that allow the inflammatory cells to enter. Dalbeth and colleagues [20], however, concluded that bone marrow infiltrates reflected by BME upregulate RANKL (receptor activator of nuclear factor kappa-B ligand) that leads to bone resorption seen in RA. Given the results of both the MRI and histologic studies, BME represents pathology that is linked to potential macroscopic destruction [20]. The reversibility of BME shown in our study, however, may indicate that this damage can be halted or repaired before macroscopic bone damage is seen on imaging. In the future, BME on MRI may prove to determine which patients are candidates for the most aggressive treatment early in disease.

Synovitis was definitely the most frequent finding in our subjects, appearing alone and with other lesions. Its ubiquity likely reflects the concept that synovitis is a primary lesion seen on MRI in RA patients. Other studies have similarly concluded that synovitis is the primary lesion in RA [21]. Synovitis resolved in two thirds of MTPs when present in isolation. Furthermore, only a very small percentage of patients developed bony lesions on subsequent studies over the 2-year period when isolated synovitis was seen at baseline, and very few bony lesions or erosions were noted on follow-up of those joints in our patients.

**Figure 2:**
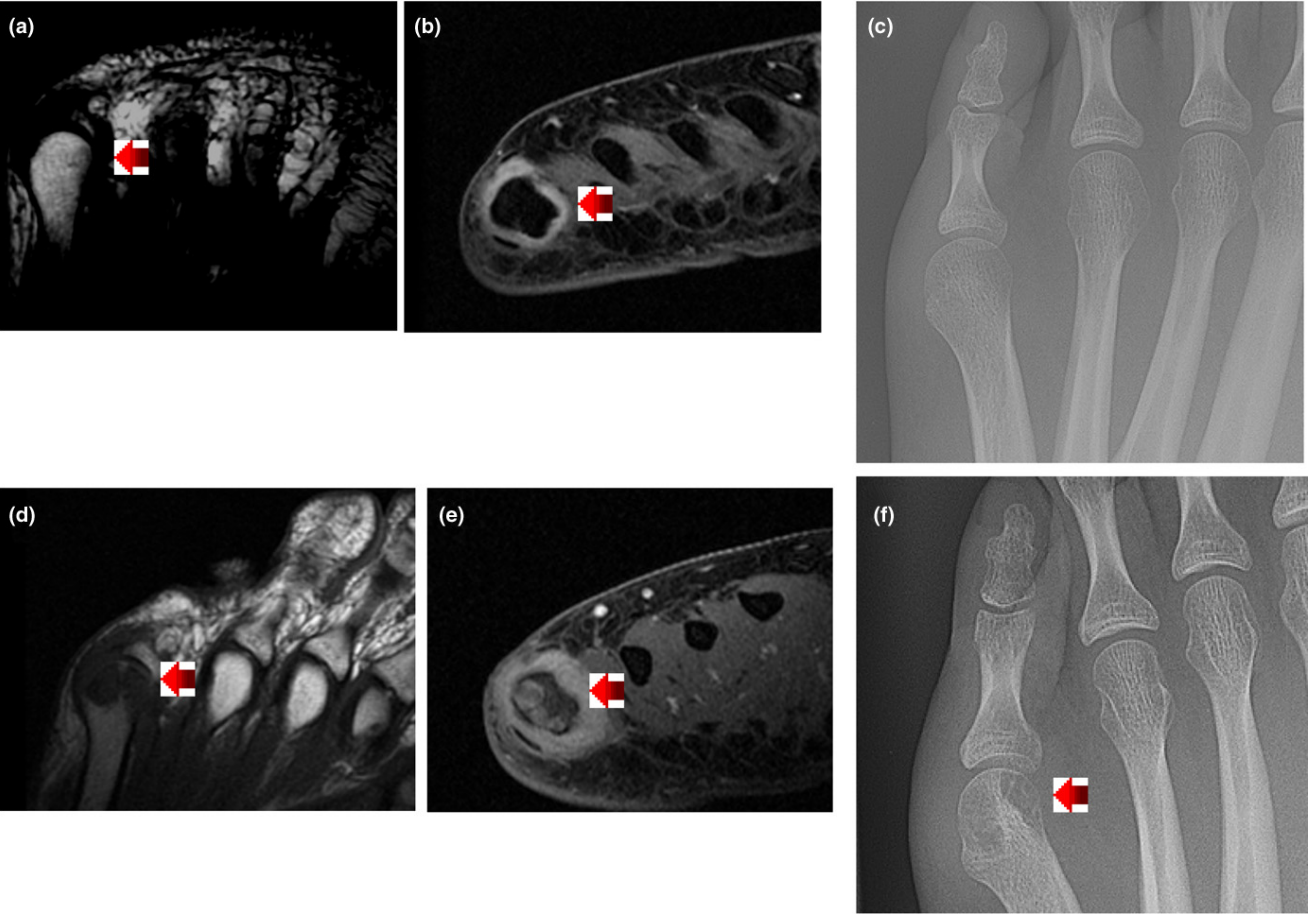
Sample imaging depicting bone defect on magnetic resonance imaging before radiographic erosion. Images **(a-c) **were taken 1 year before images **(d-f)**. Image (a) is T1-weighted and has a small defect (arrow). Image (b) is T2-weighted with fat saturation clearly showing synovitis (arrow). Image (c), the radiograph, was read as normal because the cortex was intact. Image (d), T1-weighted, clearly shows absence of bone read as erosion (arrow). Image (e) shows synovitis and bone marrow edema (arrow). Image (f) clearly shows bone erosion (arrow).

Bone defects, lesions depicting loss of trabecular bone without a cortical break, are of special interest. When new radiographic erosions occurred, the erosions not preceded by an MRI erosion were preceded by an MRI defect (example shown in Figure 2). If one generalizes both MRI erosions and defects as a bone lesion, then a bone lesion was always present prior to the development of a radiographic erosion in our study. Thus, their presence may be pathologically significant at the clinical level. Unlike MRI erosions, however, some bone defects seem reversible though not as reversible as BME or synovitis (defects reversed 37.5%, BME 70.8%, synovitis 67.3%). When this study was designed and after we collected a significant amount of data, bone defects were an official part of the RAMRIS scoring system; after this study's inception, bone defects were dropped from RAMRIS because of poor inter-reader reliability [10]. In our study, we cannot measure inter-reader reliability between our two radiologists, because they read the studies in unison and recorded their findings when they reached agreement. This is a potential weakness in the study and impacts the degree to which our findings concerning bone defects can be generalized. Nonetheless, even though the predictive value of an MRI bone lesion is low, our findings show that an MRI lesion depicting the absence of bone (either an erosion or defect) is usually evident before a radiographic erosion appears in the same joint. Thus, compared with the conclusions of future formal studies that would demand findings that are more precisely defined, our conclusions would probably be more applicable to clinical situations.

When considering all of our findings and those in other studies, one may postulate that lesions in RA progress in a certain sequence. Synovitis is a primary lesion but is highly reversible. Its presence alone does not clearly indicate that joint damage will take place. The presence of BME, on the other hand, indicates a higher potential for progression to a bone erosion and represents a critical pathologic period in the development of an RA erosion [22]. A bone erosion (and in certain cases, a bone defect) represents a more permanent lesion and is reliably present before a radiographic lesion develops. A reasonable assertion is that BME may represent the most significant period to therapeutically intervene before joint damage occurs.

## Conclusions

Our studies demonstrate that MRI erosions and BME in MTPs have a low PPV for predicting subsequent radiographic damage and a high NPV where their absence indicates a low probability of future radiographic damage (that is, if MRI reveals no MRI erosion or BME, the likelihood that an erosion will appear in that location is very low). All radiographic erosions in our cohort, however, were preceded by a bone lesion on MRI. Even though some of these lesions were defects and are no longer scored under RAMRIS, we do provide some evidence that bone defects should be considered significant at least at the clinical level. Synovitis is often transient and does not reliably lead to further damage. Because there were few radiographic erosions and a large percentage of the cohort were on anti-TNF therapy, this study suggests that modalities more sensitive than plain radiographs will likely be necessary in the biologic era to evaluate the progression of RA.

## Abbreviations

anti-CCP: anti-cyclic citrullinated peptide; BME: bone marrow edema; CI: confidence interval; CRP: C-reactive protein; DAS28: disease activity score using 28 joint counts; ESR: erythrocyte sedimentation rate; FOV: field of view; HAQ: health assessment questionnaire; HAQ-DI: health assessment questionnaire disability index; MRI: magnetic resonance imaging; MTP: metatarsophalangeal; NPV: negative predictive value; OR: odds ratio; PPV: positive predictive value; RA: rheumatoid arthritis; RAMRIS: rheumatoid arthritis magnetic resonance imaging score; RASS: rheumatoid arthritis severity scale; RF: rheumatoid factor; TE: echo time; TNF: tumor necrosis factor; TR: repetition time.

## Competing interests

The authors declare that they have no competing interests.

## Authors' contributions

MLM compiled all data, determined the analysis strategy, helped design the study, recruited patients, and wrote the manuscript and prepared its submission. PM performed the statistical analysis and wrote the statistics portion of the manuscript. DHB and JMS read all imaging and helped determine study design. DW helped design the study and recruited patients. DK helped design the study and assisted with data analysis. JL helped design the study, helped determine analysis strategy, and advised throughout the completion of the manuscript. DEF helped determine study design, recruit patients, and determine analysis strategy and advised throughout the completion of the manuscript. MHW determined study design, recruited patients, helped determine analysis strategy, advised throughout the completion of the manuscript, and approved the submitted version of the manuscript. All authors read and approved the final manuscript.

## References

[B1] Odegard S, Landewe R, Heijde D Van der, Kvien TK, Mowinckel P, Uhlig T (2006). Association of early radiographic damage with impaired physical function in rheumatoid arthritis. Arthritis Rheum.

[B2] Forslind K, Larsson EM, Johansson A, Svensson B (1997). Detection of joint pathology by magnetic resonance imaging in patients with early rheumatoid arthritis. Br J Rheumatol.

[B3] Østergaard M, Hansen M, Stoltenberg M, Jensen KE, Szkudlarek M, Pedersen-Zbinden P, Lorenzen I (2003). New radiographic bone erosions in the wrists of patients with rheumatoid arthritis are detectable with magnetic resonance imaging a median of two years earlier. Arthritis Rheum.

[B4] Ejbjerg B, Vestergaard A, Jacobsen S, Thomsen HS, Østergaard M (2005). The smallest detectable difference and sensitivity to change of magnetic resonance imaging and radiographic scoring of structural joint damage in rheumatoid arthritis finger, wrist, and toe joints. Arthritis Rheum.

[B5] Brown AK, Conaghan P, Karim Z, Quinn MA, Ikeda K, Peterfy C, Hensor E, Wakefield RJ, O'Connor P, Emery P (2008). An explanation for the apparent dissociation between clinical remission and continued structural deterioration in rheumatoid arthritis. Arthritis Rheum.

[B6] Boutry N, Larde A, Lapegue F, Solau-Gervais E, Flipo R-M, Cotten A (2003). Magnetic resonance imaging appearance of the hands and feet in patients with early rheumatoid arthritis. J Rheumatol.

[B7] Ostendorf B, Scherer A, Modder U, Schneider M (2004). Diagnostic value of magnetic resonance imaging of the forefeet in early rheumatoid arthritis when findings of the metacarpophalangeal joints of the hands remain normal. Arthritis Rheum.

[B8] Hulsmans HMJ, Jacobs JWG, Heijde DMFM van der, van Albada-Kuipers GA, Schenk Y, Bijlsma JW (2000). The course of radiologic damage during the first six years of rheumatoid arthritis. Arthritis Rheum.

[B9] Pensec VD, Saraux A, Berthelot JM, Alapetite S, Jousse S, Le Henaff C, Chales G, Thorel JB, Hoang S, Nouy-Trolle I, Martin A, Chiocchia G, Youinou P, Le Goff P (2004). Ability of foot radiographs to predict rheumatoid arthritis in patients with early arthritis. J Rheumatol.

[B10] Peterfy C, Edmonds J, Lassere M, Conaghan P, Østergaard M, McQueen FM, Genant H, Klarlund M, Ejbjerg B, Stewart N, Bird P, Shnier R, O'Connor P, Emery P (2003). OMERACT Rheumatoid arthritis MRI studies module. J Rheumatol.

[B11] Heijde D van der (1999). How to read radiographs according to the Sharp/van der Heijde method. J Rheumatol.

[B12] Mottonen TT (1988). Prediction of erosiveness and rate of development of new erosion in early rheumatoid arthritis. Ann Rheum Dis.

[B13] McQueen FM, Benton N, Crabbe J, Robinson E, Yeoman S, McClean L, Stewart N (2001). What is the fate of erosions in early rheumatoid arthritis? Tracking individual lesions using x rays and magnetic resonance imaging over the first two years of disease. Ann Rheum Dis.

[B14] Smolen J, Han C, Bala M, Maini RN, Kalden JR, Heijde D van der, Breedveld FC, Furst DE, Lipsky PE (2005). Evidence of radiographic benefit of treatment with infliximab plus methotrexate in rheumatoid arthritis patients who had no clinical improvement. Arthritis Rheum.

[B15] Yvonne PM, Goekoop-Ruiterman YPM, de Vries-Bouwstra JK, Allart CF, van Zeben D, Kerstens PJSM, Hazes MW, Zwinderman AH, Peeters AJ, de Jonge-Bok JM, Mallee C, de Beus WM, de Sonnaville PBJ, Ewals JAPM, Breedveld FC, Dijkmans BAC (2007). Comparison of treatment strategies in early rheumatoid arthritis: a randomized trial. Ann Intern Med.

[B16] McQueen FM, Benton N, Perry D, Crabbe J, Robinson E, Yeoman S, McClean L, Stewart N (2003). Bone edema scored on magnetic resonance imaging scans of the dominant carpus at presentation predicts radiographic joint damage of the hands and feet six years later in patients with rheumatoid arthritis. Arthritis Rheum.

[B17] Haavardsholm EA, Bøyesen P, Østergaard M, Schildvold A, Kvien TK (2008). Magnetic resonance imaging findings in 84 patients with early rheumatoid arthritis: bone marrow oedema predicts erosive progression. Ann Rheum Dis.

[B18] Hetland ML, Ejbjerg B, Hørslev-Petersen K, Jacobsen S, Vestergaard A, Jurik AG, Stengaard-Pedersen K, Junker P, Lottenburger T, Hansen I, Andersen LS, Tarp U, Skjødt H, Pedersen JK, Majgaard O, Svendsen AJ, Ellingsen T, Lindegaard H, Christensen AF, Vallø J, Torfing T, Narvestad E, Thomsen HS, Østergaard M, CIMESTRA study group (2009). MRI bone oedema is the strongest predictor of subsequent radiographic progression in early rheumatoid arthritis. Results from a 2-year randomised controlled trial (CIMESTRA). Ann Rheum Dis.

[B19] Jimenez-Boj E, Redlich K, Turk B, Hanslik-Schnabel B, Wanivenhaus A, Chott A, Smolen J, Schett G (2005). Interaction between synovial inflammatory tissue and bone marrow in rheumatoid arthritis. J Immunol.

[B20] Dalbeth N, Smith T, Gray S, Doyle A, Antill P, Lobo M, Robinson E, King A, Cornish J, Shalley G, Gao A, McQueen FM (2009). Cellular characterisation of magnetic resonance imaging bone oedema in rheumatoid arthritis; implications for pathogenesis of erosive disease. Ann Rheum Dis.

[B21] Conaghan P, O'Connor P, McGonagle D, Astin P, Wakefield RJ, Gibbon W, Quinn MA, Karim Z, Green M, Proudman S, Isaacs J, Emery P (2003). Elucidation of the relationship between synovitis and bone damage: a randomized magnetic resonance imaging study of individual joints in patients with early rheumatoid arthritis. Arthritis Rheum.

[B22] McQueen FM, Ostendorf B (2006). What is MRI bone oedema in rheumatoid arthritis and why does it matter?. Arthritis Res Ther.

[B23] Scheel AK, Hermann KG, Ohrndorf S, Werner C, Schirmer C, Detert J, Bollow M, Hamm B, Muller GA, Burmester GR, Backhaus M (2006). Prospective 7 year follow up imaging study comparing radiography, ultrasonography, and magnetic resonance imaging in rheumatoid arthritis finger joints. Ann Rheum Dis.

[B24] McQueen FM, Stewart N, Crabbe J, Robinson E, Yeoman S, Paul L, Tan J, McClean L (1999). Magnetic resonance imaging of the wrist in early rheumatoid arthritis reveals progression of erosions despite clinical improvement. Ann Rheum Dis.

